# The relationships between HIV stigma, emotional status, and emotional regulation among HIV-affected children in rural China

**DOI:** 10.1080/09540121.2016.1178974

**Published:** 2016-07-08

**Authors:** Wei Wei, Xiaoming Li, Sayward Harrison, Junfeng Zhao, Guoxiang Zhao

**Affiliations:** ^a^ Department of Health Promotion, Education and Behavior, Arnold School of Public Health, University of South Carolina, Columbia, SC, USA; ^b^ International Collaboration Center for Psychosocial Well-Being of Disadvantaged Children, Henan University, Kaifeng, Henan, People’s Republic of China

**Keywords:** HIV stigma, emotional status, emotional regulation, age, HIV-affected children, rural China

## Abstract

Children affected by HIV/AIDS have unique psychosocial needs that often go unaddressed in traditional treatment approaches. They are more likely than unaffected peers to encounter stigma, including overt discriminatory behaviors, as well as stereotyped attitudes. In addition, HIV-affected children are at risk for experiencing negative affect, including sadness and depression. Previous studies have identified a link between HIV stigma and the subsequent emotional status of children affected by HIV/AIDS. However, limited data are available regarding protective psychological factors that can mitigate the effects of HIV stigma and thus promote resiliency for this vulnerable population. Utilizing data from 790 children aged 6–17 years affected by parental HIV in rural central China this study aims to examine the association between HIV stigma, including both enacted and perceived stigma, and emotional status among HIV-affected children, as well as to evaluate the mediating effects of emotional regulation on the relationship between HIV stigma and emotional status. In addition, the moderating role of age is tested. Multiple regression was conducted to test the mediation model. We found that the experience of HIV stigma had a direct positive effect on negative emotions among children affected by HIV. Emotional regulation offers a level of protection, as it mediated the impact of HIV stigma on negative emotions. Moreover, age was found to moderate the relationship between perceived stigma and negative emotions. A significant interaction between perceived stigma and age suggested that negative emotions increase with age among those who perceived a higher level of stigmatization. Results suggest that children affected by HIV may benefit from interventions designed to enhance their capacity to regulate emotions and that health professionals should be aware of the link between stigma and negative emotion in childhood and adolescence and use the knowledge to inform their treatments with this population.

## Introduction

The development of increasingly effective antiretroviral medications has greatly decreased vertical transmissions of HIV (i.e., mother-to-child) in recent years, yielding a large number of children who are uninfected yet face a myriad of challenges related to their parents’ health status. HIV/AIDS is estimated to affect around 17.8 million children under 18 worldwide (UNICEF, 2013). One primary challenge faced by these children is social stigma. For the past two decades, a large body of evidence has established that people living with HIV/AIDS (PLWHA) are at risk for experiencing stigma, and that this social stigma extends to non-infected children (Chi, Li, Zhao, & Zhao, 2014). HIV-affected children may encounter stigma within extended families, schools, and communities. This stigma increases risk for poor psychological adjustment and problem behaviors, and cumulatively threatens children’s healthy development (Benetti & Kambouropoulos, 2006; Forehand et al., 1998; Pascoe & Smart Richman, 2009; Schmitt, Branscombe, Postmes, & Garcia, 2014). Although numerous studies have demonstrated that HIV stigma is significantly associated with various health indicators (e.g., Piot, Bartos, Ghys, Walker, & Schwartländer, 2001; Van Brakel, 2006), little is known about the association between HIV stigma and emotional status (i.e., the experience of positive and/or negative emotions) for children affected by HIV. This study aims to establish the relationship between HIV stigma and emotional status among the children affected by parental HIV and to examine whether emotional regulation may mediate this relationship. Furthermore, the study examines the role of age on the association between HIV stigma and emotional status.

HIV stigma is defined as the prejudice, discounting, discrediting, and discrimination that affect PLWHA and associated family members and/or friends (Parker & Aggleton, 2003). HIV stigma may take several forms, including enacted stigma (i.e., overt behaviors) and perceived stigma (i.e., awareness of stereotypes) (Herek, Gillis, & Cogan, 2009). Both types of stigma embody the social and physical rejection often faced by PLWHA and may be motivated by fear of the virus and/or moral judgment on marginalized behaviors (Chi et al., 2014; Parker & Aggleton, 2002). For HIV-affected children, stigma can be a salient and chronic stressor (Chi & Li, 2013; Paradies, 2006), and recent systematic and meta-analytic reviews have found a link between HIV stigma and negative health outcomes (e.g., depression, substance abuse) among children affected by HIV (Pantelic, Shenderovich, Cluver, & Boyes, 2015; Pascoe & Smart Richman, 2009). However, the relationship between HIV stigma and emotional status has not been well established. One previous study showed that HIV-affected children in rural China reported negative feelings such as sadness, fear, anxiety, and anger (Zhao et al., 2009). However, the role that stigma plays in the experience of negative affect for this population remains understudied and must be clarified to design effective psychosocial interventions.

Based on Watson, Clark and Tellegen’s (1988) work, emotional status can be divided into two aspects. Positive emotion refers to the extent to which an individual feels active and passionate, while negative emotion refers to feelings of distress and unpleasantness. Those two factors are used to evaluate an individual’s affective reaction to life circumstances (Werkuyten & Nekuee, 1999). A number of studies provide evidence for a strong positive relationship between negative emotion and depression (i.e., *r* = .69) and a strong negative relationship between positive emotion and depression (i.e., *r* = −.48) (Henry & Crawford, 2005; Watson & Clark, 1984). Emotional status may play a role in the development of depressive symptoms during stressful events (e.g., the experience of HIV stigma) and may be an important indicator of psychological health.

A likely mediator of the association between HIV stigma and emotional status is emotional regulation, which is the capacity to manage and regulate one’s emotions (Salovey & Mayer, 1990). Gross (1999) defines emotional regulation as “the ways individuals influence which emotions they have, when they have them, and how they experience or express these emotions” (p. 542). Emotional regulation may be most effective when individuals are able to assess their feelings accurately and adopt approaches to change negative feelings (Brackett, Palomera, Mojsa-Kaja, Reyes, & Salovey, 2010). Since emotional status is associated with depression symptoms, the capacity to manage and regulate emotions may play a role in reducing such symptoms. The experience of social stigma may decrease an individual’s self-control and emotional regulation capacity, potentially increasing unhealthy behaviors and emotional distress (Pascoe & Smart Richman, 2009). Therefore, the current study seeks to explore the role of emotional regulation as a mediator of the negative effect of HIV stigma on emotional status.

The experience of HIV stigma may differ depending upon the age of the affected child (Messer et al., 2010). However, previous studies have yielded conflicting findings as to whether younger or older children experience greater HIV stigma (Sorsdahl, Mall, Stein, & Joska, 2011; Visser & Sipsma, 2013). In addition, no studies have explored the relationship between age, HIV stigma, and emotional status. Thus, the current study examines whether age may have a moderating effect on the relationship between HIV stigma and emotional status.

The aims of the present study are to (1) investigate the association between HIV stigma and emotional status, (2) evaluate emotional regulation as a mediator of the relationship between HIV stigma and emotional status, and (3) examine age as moderator between the association of HIV stigma and emotional status among HIV-affected children in China. We hypothesize that children who encounter greater HIV stigma will experience fewer positive emotions and more negative emotions, and that emotional regulation will mediate the negative effects of HIV stigma on emotional status. We further hypothesize that age will moderate the relationship between HIV stigma and emotional status.

## Method

### Participants

The current study used baseline data from an ongoing randomized controlled trial in rural China where many residents were infected with HIV through unhygienic blood collection practices. Data were collected from a sample of 790 children 6–17 years of age. All children were orphaned by parental HIV/AIDS or lived with HIV-infected parents at the time of recruitment.

### Procedure

Recruitment and baseline data collection took place in 2012 and employed similar practices that we used in previous investigations with HIV-affected children in the central China region (see Li et al., 2009 for a thorough discussion). Children were recruited through the village social welfare system and local school systems. We accessed village-level HIV surveillance data from a rural county in central China and identified villages with the highest rates of HIV-infected individuals and/or HIV-related deaths. We then worked with local village leaders to generate lists of families caring for children affected by HIV in each village. Children with known HIV infection were not included in the study. We randomly selected families on the lists and invited one child from each family to participate in the study. Although not enumerated, the refusal rate was estimated to be less than 5%. Caregivers provided appropriate informed consent and children provided assent prior to their participation. All participants completed a survey questionnaire that asked for demographic information and included several psychosocial scales. The survey was self-administered individually or in small groups in the presence of two interviewers. For a few children (∼2% of the sample) with reading difficulties, interviewers read survey items and recorded their responses on the questionnaire. Each child received an age-appropriate gift at the completion of the study for their participation. All study procedures were approved by Institutional Review Boards at Wayne State University and Henan University.

### Measures

Scales were developed or adopted for the assessment inventory to measure variables of interest, including enacted stigma, perceived stigma, positive emotion, negative emotion, and emotional regulation. All scales were pilot tested prior to data collection and showed appropriate content validity.

Enacted stigma was measured with a 14-item scale, in which children were asked to report whether they had experienced any stigmatized actions after a parental HIV infection. Sample items included “being called bad names”, “being teased or picked on by other kids”, and “relatives stopped visiting when parents got sick or died”. Children answered items on a 5-point scale ranging from 1 (never) to 5 (always). The Cronbach’s alpha was .80 in the current study.

Perceived stigma was assessed with a 15-item scale, *Stigma Against Children Affected by AIDS* (Zhao et al., 2010). The scale assessed three dimensions of subjective awareness of social stigmatization: social exclusion (e.g., “people think children of parents living with HIV should leave their villages”), purposive avoidance (e.g., “people do not want their children to play with children of parents living with HIV”), and perceived inferiority (e.g., “people think children of parents living with HIV are unclean”). Children were asked to indicate their perception on a 4-point Likert scale (1 = strongly disagree, 4 = strongly agree). Cronbach’s alpha for the scale was .93.

To measure emotional status, a shortened version of the *Positive and Negative Affect Schedule* (*PANAS*; Watson, Clark, & Tellegen, 1988) was used. The abbreviated scale consists of 20 words describing positive or negative emotional states, for example, “excited” and “anxious”. Children were instructed to indicate to what extent they felt the particular emotion at the moment or within the past week using a 5-point Likert scale (1 = not at all, 5 = extremely). In this study, the Cronbach’s alpha was .84 for positive emotions and .81 for negative emotions.

Emotional regulation was assessed with a 6-item subscale of the social competence scale developed by Corrigan (2002). The emotional regulation subscale is used to evaluate competence of emotional adjustment (e.g., “I can control temper when I have a disagreement with my friends”). Participants answered items on a 5-point scale ranging from 0 (not at all) to 4 (very well). The Cronbach’s alpha for this scale was .80.

A mean score was calculated for items on each scale with higher scores indicating higher levels of enacted stigma, perceived stigma, positive emotions, negative emotions, and emotional regulation.

### Statistical analysis

We initially examined bivariate correlations to identify significant associations among the two types of HIV stigma, emotional status (positive and negative), and emotional regulation. Means, standard deviations, and correlations for these variables are presented in Table 1.

**Table 1.** Means, standard deviations, Cronbach’s alpha and correlations (*N* = 790).

Variable	*M* (SD)	1	2	3	4	5	Cronbach’salpha
1. Enacted stigma	1.78 (0.76)	-------					.80
2. Perceived stigma	2.77 (0.61)	.53**	-------				.93
3. Positive emotions	2.73 (0.56)	−.03	−.03	-------			.84
4. Negative emotions	2.82 (0.68)	.43**	.26**	.05	-------		.81
5. Emotional regulation	2.73 (0.56)	−.18**	−.22**	.27**	−.27**	-------	.80
6. Age	10.67 (1.79)	−.17**	−. 26**	−.04	−.11**	.05	

*p < .05.

**p < .01.

Next, to test whether emotional regulation mediates the relationship between HIV stigma and negative emotions, we performed a three-step regression analysis as recommended by Kinney (2006). The first regression model examined the relationship between HIV stigma and emotional status. The second evaluated whether there was a significant association between HIV stigma and emotional regulation. The final regression determined whether the effect of HIV stigma was reduced when the mediator was introduced into the model predicting emotional status. The significance of estimated mediation effects was assessed with Sobel test using the bootstrapping method (Preacher & Hayes, 2008).

Finally, in order to examine the potential moderating effect of age on emotional status, hierarchical multiple regression analyses were conducted for positive emotions and negative emotions with three steps as recommend by Baron and Kenny (1986). In each hierarchical regression model, the variable order of entry was as follows: at step 1, the independent variable (HIV stigma) was entered into the regression equation; at step 2, the moderator (age) was entered into the regression equation; and at step 3 the interaction between HIV stigma and age was added.

## Results

### Preliminary analyses

Demographic characteristics of the participants (*N* = 790) including gender, age, and ethnicity were analyzed. The sample consisted of 51.6% boys (*n* = 408) and 48.4% girls (*n* = 382). Average age of participants was 10.51 years (*SD* = 1.99) with a range of 6–17 years. The majority (97%) of participants were of Han ethnicity, the predominant ethnic group in China.

As shown in Table 1, enacted stigma was positively associated with negative emotions (*r* = .40, *p* < .01), but not with positive emotions. Perceived stigma was also positively related to negative emotions (*r* = .26, *p* < .01), but not positive emotions. Emotional regulation was positively related to positive emotions (*r* = .27, *p* < .01), but negatively related to enacted stigma (*r* = −.18, *p* < .01), perceived stigma (*r* = −.22, *p* < .01), and negative emotions (*r* = −.27, *p* < .01). Because no correlation exceeded 0.70, the assumption of multicollinearity was not violated (Kline, 2005).

**Figure 1.**
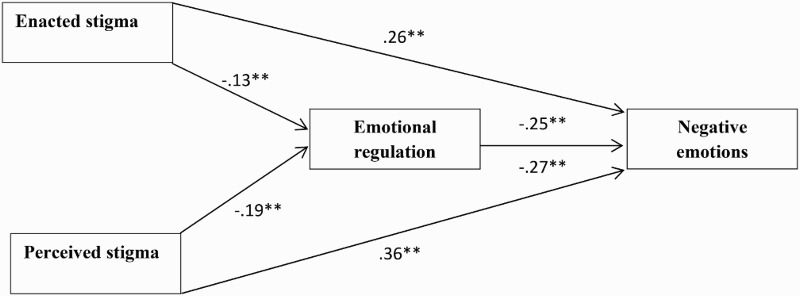
Final path model showing the indirect effect of both enacted and perceived stigma on negative emotions via emotional regulation. * *p* < .05, ** *p* < .01.

Multiple linear regressions demonstrated that emotional regulation partially mediated the relationship between negative emotions and both types of stigma (Figure 1), including enacted stigma (indirect effect = .032, 95% CI [0.01, 0.05]) and perceived stigma (indirect effect = .05, 95% CI [0.02, 0.08]). Moreover, enacted stigma was significantly associated with negative emotions (*β* = .26, *p* < .01) and perceived stigma showed significant association with negative emotions (*β* = .36, *p* < .01). Both enacted and perceived stigma had no significant association with positive emotions. Emotional regulation was significantly associated with both positive emotions (*β* = .33, *p* < .01) and negative emotions (*β* = −.27, *p* < .01). As shown in Table 2, for each of the models, the Sobel test using the bootstrapping method indicated significant mediation.

**Table 2.** Mediation analyses and indirect effects and 95% confidence intervals with 1000 bootstrap resamples using the Sobel test (*N* = 790).

DV = Negative emotions IVs	*β*	*T*	Boot SE	Indirect effect	Boot LLCI	Boot ULCI
Enacted stigma	.26	11.2**				
*Emotional regulation*^a^	−.25	−6.4**	0.01	0.05**	0.02	0.08
Perceived stigma	.36	6.1**				
*Emotional regulation*^b^	−.27	−6.5**	0.01	0.03**	0.01	0.05

^a^Indirect effect on the relationship between negative emotions and enacted stigma.

^b^Indirect effect on the relationship between negative emotions and perceived stigma.

*p < .05.

**p < .01.

### Moderation effects

**Figure 2.**
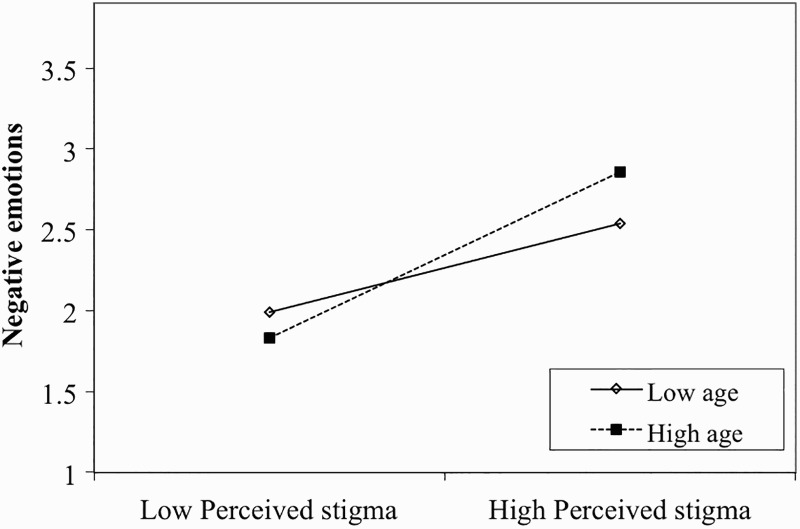
Interaction of perceived stigma and age on negative emotions scores. Note: Low age = 1 SD below mean; high age = 1 SD above mean.

As shown in Table 3, the perceived stigma × age interaction term was significant for negative emotions for the total sample (*β* = .04, *p* < .05, Δ*R^2^* = 0.01), with higher age associated with greater experience of negative emotions. This suggests that the effect of perceived stigma on negative emotions is moderated by age in HIV-affected children. To illustrate the effect of age, we examined the simple slope of the regression using the high (one standard deviation above the mean) and low (one standard deviation below the mean) values for age. The method follows the procedures outlined by Hayes and Matthes (2009). As illustrated in Figure 2, there was a significant relationship between perceived stigma and negative emotions at younger age [*β* = .18, *t*(769) = 3.25, *p* < .01] and at older age [*β* = .34, *t*(769) = 6.77, *p* < .01].

**Table 3.** Hierarchical regression analysis results for the moderating effect of perceived stigma and age on negative emotions (*N* = 790).

Variables	*β*	*t*	*R*^2^	Δ*R*^2^
Negative emotions			0.08**	
Perceived stigma	.26	7.5**		0.07**
Age	−.02	−1.0		0.002
Perceived stigma × Age	.04	2.4*		0.01*

*p < .05.

**p < .01

## Discussion

Prior research has documented the negative effects of HIV stigma on numerous health indicators for PLWHA, including symptoms of depression (Forehand et al., 1998). However, limited research has examined the relationship between HIV stigma and emotional status for children affected by HIV or explored potential resiliency factors that could prevent the negative effects of stigmatizing experiences. In this study, we examined the relationships among two types of HIV stigma (i.e., enacted stigma and perceived stigma), emotional status (i.e., positive emotions and negative emotions), emotional regulation, and age in a sample of HIV-affected children in rural China. Both perceived stigma and enacted stigma were positively associated with negative emotions, but not with positive emotions. This is consistent with previous findings that those who perceive greater stigma also experience more intense negative emotions (Werkuyten & Nekuee, 1999; Wills, 1986). Our findings extend the work of Zhao et al., (2009), confirming that HIV-related stigma appears to impact not only PLWHA but also their children. The negative attitudes and overt hostile acts experienced by these children likely contribute to their increased risk for sadness and other depressive symptoms.

This is one of the first studies to our knowledge to investigate the mediating role of emotional regulation on the relationship between HIV stigma and emotional status for children affected by HIV. Our findings confirm that emotional regulation may reduce and change the expression of negative emotions (Brackett et al., 2010), and extend these previous findings to HIV-affected children. Emotional regulation may be an effective buffer to reduce the negative effects of HIV stigma. This may be particularly important for HIV-affected children who face challenges that are often outside of their control (e.g., parental illness, changes in guardianship). Emotional regulation may enable them to adapt to such changes in a resilient manner. Our results provide preliminary evidence that psychosocial interventions for HIV-affected children may be made more effective by including components that teach emotional regulation skills. For instance, interventions may wish to include components on recognizing and labeling emotions; understanding emotional and physical reactions to daily life circumstances; developing plans for effective coping; and seeking emotional support from others. Several such “coping skill” interventions are already in existence. However, for them to be effective for this population, preexisting interventions will likely need to be adapted to the particular culture context of the target audience and modified to specifically address the unique challenges (i.e., enacted and perceived HIV stigma) that children affected by HIV are likely to encounter.

The current study also presents new findings on the role of age in moderating the relationship between perceived stigma and negative emotions for HIV-affected children. We found that age influenced the association between perceived stigma and negative emotions for both older and younger children, suggesting that the relationship between perceived stigma and negative emotions may persist throughout childhood and adolescence. In addition, we found that the impact of perceived stigma on negative emotions increased with age. As children age and their cognitive and social-emotional skills increase, they may begin to better understand the ways they and their families are discriminated against (Rankin, Lane, Gibbons, & Gerrard, 2004). Adolescents may also be more sensitive to feelings of social prejudice, rejection, and isolation that they encounter within their social contexts. This suggests that psychosocial interventions should be attentive to the ages of their target population, and may wish to include components for teenagers to help them manage negative emotions, cope with stigmatizing events, and understand how these two experiences may be linked. Future research should further explore the complex relationship between HIV stigma, negative emotions, and age, as well as how these experiences vary according to HIV stigma type and base level of negative emotion.

## Limitations and conclusion

The current study has several limitations. First, data were gathered in 2012 at baseline of a longitudinal intervention trial. While we do not expect the variables of interest to have changed significantly since then, the age of the data could be a potential limitation. Second, results are exploratory in nature, and future research should use a longitudinal approach to examine causal relationships among the variables of interest. In addition, the study sample may not be representative of all children affected by HIV/AIDS. Children included in the study resided in rural, central China where the HIV epidemic has primarily been caused by unhygienic blood collection. Children’s experience of stigma may potentially differ depending upon culture, source of infection, and a host of other factors.

Despite these limitations, the current study provides preliminary evidence that the impact of HIV stigma on negative emotions could potentially be reduced by emotional regulation. Given these findings, intervention strategies that seek to enhance the capacity of children to regulate their emotions and manage maladaptive cognitions may lessen the impact of HIV stigma. In addition, the study suggests that the strength of the relationship between perceived stigma and negative emotions may differ by age, making it important for psychosocial interventions to modify programming depending on the age of the target population. Future studies should continue to explore the multi-faceted emotional and social experiences that stigma and discrimination bring about in order to promote resilience and well-being throughout childhood and adolescence for those affected by HIV/AIDS.

## Disclosure statement

No potential conflict of interest was reported by the authors.
